# Multiallelic epistatic model for an out-bred cross and mapping algorithm of interactive quantitative trait loci

**DOI:** 10.1186/1471-2229-11-148

**Published:** 2011-10-31

**Authors:** Chunfa Tong, Bo Zhang, Zhong Wang, Meng Xu, Xiaoming Pang, Jingna Si, Minren Huang, Rongling Wu

**Affiliations:** 1 The Key Laboratory of Forest Genetics and Gene Engineering, Nanjing Forestry University, Nanjing, Jiangsu 210037, China; 2 Center for Statistical Genetics, The Pennsylvania State University, Hershey, PA 17033, USA; 3 Center for Computational Biology, National Engineering Laboratory for Tree Breeding, Key Laboratory of Genetics and Breeding in Forest Trees and Ornamental Plants, Beijing Forestry University, Beijing 100083, China

## Abstract

**Background:**

Genetic mapping has proven to be powerful for studying the genetic architecture of complex traits by characterizing a network of the underlying interacting quantitative trait loci (QTLs). Current statistical models for genetic mapping were mostly founded on the biallelic epistasis of QTLs, incapable of analyzing multiallelic QTLs and their interactions that are widespread in an outcrossing population.

**Results:**

Here we have formulated a general framework to model and define the epistasis between multiallelic QTLs. Based on this framework, we have derived a statistical algorithm for the estimation and test of multiallelic epistasis between different QTLs in a full-sib family of outcrossing species. We used this algorithm to genomewide scan for the distribution of mul-tiallelic epistasis for a rooting ability trait in an outbred cross derived from two heterozygous poplar trees. The results from simulation studies indicate that the positions and effects of multiallelic QTLs can well be estimated with a modest sample and heritability.

**Conclusions:**

The model and algorithm developed provide a useful tool for better characterizing the genetic control of complex traits in a heterozygous family derived from outcrossing species, such as forest trees, and thus fill a gap that occurs in genetic mapping of this group of important but underrepresented species.

## Background

Approaches for quantitative trait locus (QTL) mapping were developed originally for experimental crosses, such as the backcross, double haploid, RILs or F_2_, derived from inbred lines [1-3]. Because of the homozygosity of inbred lines, the Mendelian (co)segregation of all markers each with two alternative alleles in such crosses can be observed directly. In practice, there is also a group of species of great economical and environmental importance - out-crossing species, such as forest trees, in which traditional QTL mapping approaches cannot be appropriately used. For these species, it is difficult or impossible to generate inbred lines due to long generation intervals and high heterozygosity [4], although experimental hybrids have been commercially used in practical breeding programs.

For a given outbred line, some markers may be heterozygous, whereas others may be homozygous over the genome. All markers may, or may not, have the same allele system between any two outbred lines used for a cross. Also, for a pair of heterozygous loci, their allelic configuration along two homologous chromosomes (i.e., linkage phase) cannot be observed from the segregation pattern of genotypes in the cross [5,6]. Unfortunately, a consistent number of alleles across different markers and their known linkage phases are the prerequisites for statistical mapping approaches described for the backcross or F_2_. Grattapaglia and Sederoff [7] proposed a so-called pseudo-test backcross strategy for linkage mapping in a controlled cross between two outbred parents. This strategy is powerful for the linkage analysis of those testcross markers that are heterozygous in one parent and null in the other, although it fails to consider many other marker cross types, such as intercross markers and dominant markers, that occur for an outbred cross. Maliepaard et al. [8] derived numerous formulas for estimating the linkage between different types of markers by correctly determining the linkage phase of markers. A general model has been developed for simultaneous estimation of the linkage and linkage phase for any marker cross type in outcrossing populations [9,10]. Stam [11] wrote powerful software for integrating genetic linkage maps using different types of markers.

Statistical methods for QTL mapping in a full-sib family of outcrossing species have not received adequate attention. Lin et al. [12] developed a model that takes into account uncertainties about the number of alleles across the genome. Wu et al. [13] used this model to reanalyze a full-sib family data for poplar trees [14], leading to the detection of new QTLs for biomass traits which were not discovered by traditional approaches. With increasing recognition of the role of epistasis in controlling and maintaining quantitative variation [15], it is crucial to extend Lin et al.'s model to map the epistatic of QTLs by which to elucidate a detailed and comprehensive perspective on the genetic architecture of a quantitative trait. However, the well-established theory and model for epistasis are mostly based on biallelic genes [16] and their estimation and test are made for a pedigree derived from inbred lines [17]. Until now, no models and algorithms have been available for characterizing the epistasis of multiallelic QTLs in an outcrossing population.

In this article, we will extend the theory for biallelic epistasis to model the epistasis between different QTLs each with multiple alleles. The multiallelic epistatic theory is then implemented into a statistical model for QTL mapping based on a mixture model. We have derived a closed form for the estimation of the main and interactive effects of multiallelic QTLs within the EM framework. Our model allows geneticists to test the effects of individual genetic components on trait variation. The estimating model has been investigated through simulation studies and validated by an example of QTL mapping for poplar trees [18]. The algorithm has been packed to a newly developed package of software, 3FunMap, derived to map QTLs in a full-sib family [19].

## Quantitative Genetic Model

### Additive-dominance Model

Randomly select two heterozygous lines as parents P_1 _and P_2 _to produce a full-sib family, in which a QTL will form four genotypes if the two lines have completely different allele systems. Let *μ*_*uv *_be the value of a QTL genotype inheriting allele *u *(*u = *1,2) from parent P_1 _and allele *v *(*v *= 3, 4) from parent P_2_. Based on quantitative genetic theory, this genotypic value can be partitioned into the additive and dominant effects as follows:

(1) $$\mu_{uv}=\mu +\alpha_{u}+\beta_{v}+\gamma_{uv},$$

where *μ *is the overall mean, *α*_*u *_and *β*_*v *_are the allelic (additive) effects of allele *u *and *v*, respectively, and *γ*_*uv *_is the interaction (dominant) effect at the QTL. Considering all possible alleles and allele combinations between the two parent, there are a total of four additive effects (*α*_1 _and *α*_2 _from parent P_1 _and *β*_3 _and *β*_4 _from parent P_2 _and four dominant effects (*γ*_13_, *γ*_14_, *γ*_23 _and *γ*_34_). But these additive and dominant effects are not independent and, therefore, are not estimable. After parameterization, there are two independent additive effects, *α *= *α*_1 _= -*α*_2 _and *β*_3 _= *β*_3 _= -*β*_4_, and one dominant effect, *γ *= *γ*_13 _= -*γ*_14 _= -*γ*_23 _= *γ*_24_, to be estimated.

Let **u **= (*μ*_*uv*_)_4 × 1 _and **a **= (*μ, α, β, γ*)^T^, which can be connected by a design matrix **D**. We have

u=Da,

where

$$D=\left[\begin{array}{cccc} 1 & 1 & 1 & 1\\ 1 & 1 & -1 & -1\\ 1 & -1 & 1 & -1\\ 1 & -1 & -1 & 1 \end{array}\right].$$

The expression of a can be obtained from the expression of **u **by

(2) $$a=D^{-1}u.$$

### Additive-dominance-epistatic Model

If there are two segregating QTL in the full-sib family, the epistatic effects due to their nonallelic interactions should be considered. The theory for epistasis in an inbred family [16] can be readily extended to specify different epistatic components for outbred crosses. Consider two epistatic multiallelic QTL, each of which has four different genotypes, 13, 14, 23, and 24, in the outbred progeny. Let $\mu_{u_{1}v_{1}∕u_{2}v_{2}}$ be the genotypic value for QTL genotype *u*_1_*v*_1_/*u*_2_*v*_2 _for *u*_1_,*u*_2 _= 1,2 and *v*_1_,*v*_2 _= 3,4 and $u=\left(\mu_{u_{1}v_{1}∕u_{2}v_{2}}\right)$ be the corresponding mean vector. The two-QTL genotypic value is partitioned into different components as follows:

(3) $$\begin{array}{ll} \mu_{u_{1}v_{1}∕u_{2}v_{2}} & =\mu +\alpha_{1}+\beta_{1}+\gamma_{1}+\alpha_{2}+\beta_{2}+\gamma_{2}\;\\ \; & +I_{\alpha \alpha }+I_{\alpha \beta }+I_{\beta \alpha }+I_{\beta \beta }+J_{\alpha \gamma }+J_{\beta \gamma }+K_{\gamma \alpha }+K_{\gamma \beta }+L_{\gamma \gamma }\;\\ & \; \end{array}$$

where

(1) *μ *is the overall mean;

(2) *α*_1 _is the additive effect due to the substitution from allele 1 to 2 at the first QTL;

(3) *β*_1 _is the additive effect due to the substitution from allele 3 to 4 at the first QTL;

(4) *γ*_1 _is the dominant effect due to the interaction between alleles from different parents;

(5) *α*_2 _is the additive effect due to the substitution from allele 1 to 2 at the second QTL;

(6) *β*_2 _is the additive effect due to the substitution from allele 3 to 4 at the second QTL;

(7) *γ*_2 _is the dominant effect due to the interaction between alleles from different parents;

(8) *I*_*αα *_is the additive × additive epistatic effect due to the interaction between the substitutions from allele 1 to 2 at the first and second QTLs;

(9) *I*_*αβ *_is the additive × additive epistatic effect due to the interaction between the substitutions from allele 1 to 2 at the first QTL and from allele 3 to 4 at the second QTL;

(10) *I*_*βα *_is the additive × additive epistatic effect due to the interaction between the sub-stitutions from allele 3 to 4 at the first QTL and from allele 1 to 2 at the second QTL;

(11) *I*_*αβ *_is the additive × additive epistatic effect due to the interaction between the sub-stitutions from allele 3 to 4 at the first and second QTLs;

(12) *J*_*αγ *_is the additive × dominant epistatic effect due to the interaction between the substitutions from allele 1 to 2 at the first QTL and the dominant effect at the second QTL;

(13) *J*_*βγ *_is the additive × dominant epistatic effect due to the interaction between the substitutions from allele 3 to 4 at the first QTL and the dominant effect at the second QTL;

(14) *K*_*γα *_is the dominant × additive epistatic effect due to the interaction between the dominant effect at the first QTL and the substitutions from allele 1 to 2 at the second QTL;

(15) *K*_*γβ *_is the dominant × additive epistatic effect due to the interaction between the dominant effect at the first QTL and the substitutions from allele 3 to 4 at the second QTL;

(16) *L*_*γγ *_is the dominant × dominant epistatic effect due to the interaction between the dominant effects at the first and second QTLs.

Genetic effect parameters for two interacting QTL are arrayed in **a **= (*μ, α*_1_, *β*_1_, *γ*_1_, *α*_2_, *β*_2_, *γ*_2_, *I*_*αα*_, *I*_*αβ*_, *I*_*βα*_, *I*_*ββ*_, *J*_*αγ*_, *J*_*βγ*_, *K*_*γα*_, *K*_*γβ*_, *L*_*γγ*_)^T^. We relate the genotypic value vector and genetic effect vector by

u=Da,

where design matrix

$$D=\left[\begin{array}{cccccccccccccccc} 1 & 1 & 1 & 1 & 1 & 1 & 1 & 1 & 1 & 1 & 1 & 1 & 1 & 1 & 1 & 1\\ 1 & 1 & 1 & 1 & 1 & -1 & -1 & 1 & -1 & 1 & -1 & -1 & -1 & 1 & -1 & -1\\ 1 & 1 & 1 & 1 & -1 & 1 & -1 & -1 & 1 & -1 & 1 & -1 & -1 & -1 & 1 & -1\\ 1 & 1 & 1 & 1 & -1 & -1 & 1 & -1 & -1 & -1 & -1 & 1 & 1 & -1 & -1 & 1\\ 1 & 1 & -1 & -1 & 1 & 1 & 1 & 1 & 1 & -1 & -1 & 1 & -1 & -1 & -1 & -1\\ 1 & 1 & -1 & -1 & 1 & -1 & -1 & 1 & -1 & -1 & 1 & -1 & 1 & -1 & 1 & 1\\ 1 & 1 & -1 & -1 & -1 & 1 & -1 & -1 & 1 & 1 & -1 & -1 & 1 & 1 & -1 & 1\\ 1 & 1 & -1 & -1 & -1 & -1 & 1 & -1 & -1 & 1 & 1 & 1 & -1 & 1 & 1 & -1\\ 1 & -1 & 1 & -1 & 1 & 1 & 1 & -1 & -1 & 1 & 1 & -1 & 1 & -1 & -1 & -1\\ 1 & -1 & 1 & -1 & 1 & -1 & -1 & -1 & 1 & 1 & -1 & 1 & -1 & -1 & 1 & 1\\ 1 & -1 & 1 & -1 & -1 & 1 & -1 & 1 & -1 & -1 & 1 & 1 & -1 & 1 & -1 & 1\\ 1 & -1 & 1 & -1 & -1 & -1 & 1 & 1 & 1 & -1 & -1 & -1 & 1 & 1 & 1 & -1\\ 1 & -1 & -1 & 1 & 1 & 1 & 1 & -1 & -1 & -1 & -1 & -1 & -1 & 1 & 1 & 1\\ 1 & -1 & -1 & 1 & 1 & -1 & -1 & -1 & 1 & -1 & 1 & 1 & 1 & 1 & -1 & -1\\ 1 & -1 & -1 & 1 & -1 & 1 & -1 & 1 & -1 & 1 & -1 & 1 & 1 & -1 & 1 & -1\\ 1 & -1 & -1 & 1 & -1 & -1 & 1 & 1 & 1 & 1 & 1 & -1 & -1 & -1 & -1 & 1 \end{array}\right].$$

Thus, the genetic effect vector can be expressed, in terms of the genotypic value vector, as

(4) $$a=D^{-1}1u.$$

If we have alleles 1 = 3 and 2 = 4 for an outbred family, Equations 1 and 3 will be reduced to traditional biallelic additive-dominant and biallelic additive-dominant-epistatic genetic models, respectively [20].

## Statistical Model

### Likelihood

Suppose there is a full-sib family of size *n *derived from two outbred lines. Consider two interacting QTLs for a quantitative trait. Let *u*_1_*v*_1 _and *u*_2_*v*_2 _denote a general genotype at QTL 1 and 2, respectively, where *u*_1 _and *u*_2 _(*u*_1_,*u*_2 _= 1,2) are the alleles inherited from parent P_1 _and *v*_1 _and *v*_2 _(*v*_1_,*v*_2 _= 3,4) are the alleles inherited from parent P_2_. The linear model of the trait value (*y*_*i*_) for individual *i *affected by the two QTLs is written as

(5) $$y_{i}=\overset{2}{\underset{u_{1}=1}{\sum }}\overset{4}{\underset{v_{1}=3}{\sum }}\overset{2}{\underset{u_{2}=1}{\sum }}\overset{4}{\underset{v_{2}=3}{\sum }}\xi_{iu_{1}v_{1}∕u_{2}v_{2}}\mu_{u_{1}v_{1}∕u_{2}v_{2}}+ei,$$

where $\xi_{iu_{1}v_{1}∕u_{2}v_{2}}$ is the indicator variable for QTL genotypes defined as 1 if a particular genotype *u*_1_*v*_1_/*u*_2_*v*_2 _is considered for individual *i *and 0 otherwise, and *e*_*i *_is the residual error normally distributed with mean 0 and variance *σ*^2^. The probability with which individual *i *carries QTL genotype *u*_1_*v*_1_/*u*_2_*v*_2 _can be inferred from its marker genotype, with this conditional probability expressed as $\omega_{u_{1}v_{1}∕u_{2}v_{2}|i}$[20].

The log-likelihood of the putative QTLs given the trait value (*y*) and marker information (M) is given by

(6) $$L\left(\Theta |y,M\right)=\overset{n}{\underset{i=1}{\prod }}\overset{2}{\underset{u_{1}=1}{\sum }}\overset{4}{\underset{v_{1}=3}{\sum }}\overset{2}{\underset{u_{2}=1}{\sum }}\overset{4}{\underset{v_{2}=3}{\sum }}\omega_{u_{1}v_{1}∕u_{2}v_{2}|i}f_{u_{1}v_{1}∕u_{2}v_{2}}\left(y_{i}\right),$$

where Θ is the vector for unknown parameters that include the QTL position expressed by the conditional probabilities $\left(\omega_{u_{1}v_{1}∕u_{2}v_{2}|i}\right)$, QTL genotypic values $\left(\mu_{u_{1}v_{1}∕u_{2}v_{2}}\right)$ and the residual variance (*σ*^2^). The first parameters, denoted by Θ_*p*_, are contained in the mixture proportions of the above model, whereas the second two, denoted by Θ_*q*_, are quantitative genetic parameters. Normal distribution density $fu_{1}v_{1}∕u_{2}v_{2}\left(y_{i}\right)$ has mean $\mu_{u_{1}v_{1}∕u_{2}v_{2}}$ and variance *σ*^2^.

### EM Algorithm

The standard EM algorithm is developed to obtain the estimates of the unknown vector. By differentiating the log-likelihood of equation (6) with respect to two groups of unknown parameters (Θ_*p*_, Θ_*q*_), we have

∂∂ΘlogL(Θ|y,M)= ∑i=1n∑u1=12∑v1=34∑u2=12∑v2=34fu1v1∕u2v2(yi)∂∂Θpωu1v1∕u2v2|i+ωu1v1∕u2v2|i∂∂Θqfu1v1u2v2(yi)∑u′1=12∑v′1=34∑u′2=12∑v′2=34ωu′1v′1∕u′2v′2|ifu′1v′1∕u′2v′2(yi)= ∑i=1n∑u1=12∑v1=34∑u2=12∑v2=34ωu1v1∕u2v2|ifu1v1∕u2v2(yi)1ωu1v1∕u2v2|i∂∂Θpωuv|i∑u′1=12∑v′1=34∑u′2=12∑v′2=34ωu′1v′1∕u′2v′2|ifu′1v′1∕u′2v′2(yi)+ωu1v1∕u2v2|ifu1v1∕u2v2(yi)∂∂Θq logfu1v1∕u2v2(yi)∑u′1=12∑v′1=34∑u′2=12∑v′2=34ωu′1v′1∕u′2v′2|ifu′1v′1∕u′2v′2(yi)= ∑i=1n∑u1=12∑v1=34∑u2=12∑v2=34∏u1v1∕u2v2|i1ωu1v1∕u2v2|i∂∂Θpωu1v1∕u2v2|i+∂∂Θq logfu1v1∕u2v2(yi),

where we define

(7) $$\prod_{u_{1}v_{1}∕u_{2}v_{2}|i}=\frac{\omega_{u_{1}v_{1}∕u_{2}v_{2}|i}\;f_{u_{1}v_{1}∕u_{2}v_{2}}\left(yi\right)}{\sum_{{u^{\prime }}_{1}=1}^{2}\sum_{{v^{\prime }}_{1}=3}^{4}\sum_{{u^{\prime }}_{2}=1}^{2}{\sum_{{v^{\prime }}_{2}=3}^{4}\omega_{{u^{\prime }}_{1}{v^{\prime }}_{1}∕{u^{\prime }}_{2}{v^{\prime }}_{2}|i\;}f}_{{u^{\prime }}_{1}{v^{\prime }}_{1}∕{u^{\prime }}_{2}{v^{\prime }}_{2}}\left(y_{i}\right)}$$

which could be thought of as a posterior probability that individual *i *has a QTL genotype *u*_1_*v*_1_/*u*_2_*v*_2_.

In the E step, calculate the posterior probabilities of QTL genotypes given the marker genotype of individual *i *by equation (7). In the M step, estimate the maximum likelihood estimates (MLEs) of the unknown parameters by solving $\frac{\partial }{\partial \Theta }\log L\left(\Theta |y,M\right)=0.$. The closed forms for estimating the genotypic values and residual variance are derived as

(8) $$\begin{array}{ll} {\hat{\mu }}_{u_{1}v_{1}∕u_{2}v_{2}} & =\frac{\sum_{u_{1}=1}^{2}\sum_{v_{1}=3}^{4}\sum_{u_{2}=1}^{2}\sum_{v_{2}=3}^{4}\prod_{u_{1}v_{1}∕u_{2}v_{2}|i}y_{i}}{\sum_{u_{1}=1}^{2}\sum_{v_{1}=3}^{4}\sum_{u_{2}=1}^{2}\sum_{v_{2}=3}^{4}\prod_{u_{1}v_{1}∕u_{2}v_{2}|i}}\;\\ {\hat{\sigma }}^{2} & =\frac{1}{n}\overset{n}{\underset{i=1}{\sum }}\overset{2}{\underset{u_{1}=1}{\sum }}\overset{4}{\underset{v_{1}=3}{\sum }}\overset{2}{\underset{u_{2}=1}{\sum }}\overset{4}{\underset{v_{2}=3}{\sum }}{\left(y_{i}{\hat{\mu }}_{u_{1}v_{1}∕u_{2}v_{2}}\right)}^{2}\prod_{u_{1}v_{1}∕u_{2}v_{2}|i\cdot }\;\\ & \; \end{array}$$

By giving initial values for the parameters, the E and M steps are iterated until the estimates are stable. The stable values are the MLEs of the unknown parameters. Note that the QTL position within a marker interval can be estimated by treating the position is fixed. Using a grid search, we can obtain the MLE of the QTL position from the peak of the profile of the log-likelihood ratio test statistics across a chromosome.

## Hypothesis Tests

After the parameters are estimated, a number of hypothesis tests can be made. The existence of a QTL can be tested by formulating the null hypothesis expressed as

(9) $$\begin{array}{c} H_{0}\;:\;\mu_{u_{1}v_{1}∕u_{2}v_{2}}\equiv \mu ,\text{for}\;u_{1},v_{1}=1,2\;\text{and}\;u_{2},v_{2}=3,4\\ H_{1}:\;\text{at}\;\text{least}\;\text{one}\;\text{of}\;\text{the}\;\text{equalities}\;\text{above}\;\text{does}\;\text{not}\;\text{hold}. \end{array}$$

The likelihoods under the reduced (*H*_0_) and full model (*H*_1_) are calculated and their log-likelihood ratio (LR) is then estimated by

(10) $$LR=-2\;\text{In}\left[\frac{{\text{L}}_{\text{0}}\left({\tilde{\Theta }}_{p},{\tilde{\Theta }}_{q}|y\right)}{L_{0}\left({\hat{\Theta }}_{p},{\hat{\Theta }}_{q}|y,M\right)}\right],$$

where the tildes and hats are the MLEs under the *H*_0 _and *H*_1_, respectively. The critical threshold for declaring the existence of a QTL can be empirically determined from permutation tests [21].

Hypothesis tests for different genetic effects including the additive (*α*_1_, *β*_1_, *α*_2_, *β*_2_), dominant (*γ*_1_, *γ*_2_) and additive × additive (*I*_αα_, *I*_*αβ*_, *I*_*βα*_, *I*_*ββ*_), additive × dominant (*J*_*αγ*_, *J*_*βγ*_), dominant × additive (*K*_*γα*_, *K*_*γβ*_) and dominant × dominant (*L*_*γγ*_) epistatic effects can be formulated, with the respective null hypotheses:

Under each null hypothesis, the genotypic values should be constrained by

(11) $$\begin{array}{c} \mu_{13∕13}+\mu_{13∕14}+\mu_{13∕23}+\mu_{13∕24}+\mu_{14∕13}+\mu_{14∕14}+\mu_{14∕23}+\mu_{14∕24}\\ =\mu_{23∕13}+\mu_{23∕14}+\mu_{23∕23}+\mu_{23∕24}+\mu_{24∕13}+\mu_{24∕14}+\mu_{24∕23}+\mu_{24∕24} \end{array}$$

for *H*_0 _: *α*_1 _= 0,

(12) $$\begin{array}{c} \mu_{13∕13}+\mu_{13∕14}+\mu_{13∕23}+\mu_{13∕24}+\mu_{23∕13}+\mu_{23∕14}+\mu_{23∕23}+\mu_{23∕24}\\ =\mu_{14∕13}+\mu_{14∕14}+\mu_{14∕23}+\mu_{14∕24}+\mu_{24∕13}+\mu_{24∕14}+\mu_{24∕23}+\mu_{24∕24} \end{array}$$

for *H*_0 _: *β*_1 _= 0,

(13) $$\begin{array}{c} \mu_{13∕13}+\mu_{13∕14}+\mu_{14∕13}+\mu_{14∕14}+\mu_{23∕13}+\mu_{23∕14}+\mu_{24∕13}+\mu_{24∕14}\\ =\mu_{13∕23}+\mu_{13∕24}+\mu_{14∕23}+\mu_{14∕24}+\mu_{23∕23}+\mu_{23∕24}+\mu_{24∕23}+\mu_{24∕24} \end{array}$$

for *H*_0 _: *α*_2 _= 0,

(14) $$\begin{array}{c} \mu_{13∕13}+\mu_{13∕23}+\mu_{14∕13}+\mu_{14∕23}+\mu_{23∕13}+\mu_{23∕23}+\mu_{24∕13}+\mu_{24∕23}\\ =\mu_{13∕14}+\mu_{13∕24}+\mu_{14∕14}+\mu_{14∕24}+\mu_{23∕14}+\mu_{23∕24}+\mu_{24∕14}+\mu_{24∕24}, \end{array}$$

for *H*_0 _: *β*_2 _= 0,

(15) $$\begin{array}{c} \mu_{13∕13}+\mu_{13∕14}+\mu_{13∕23}+\mu_{13∕24}+\mu_{24∕13}+\mu_{24∕14}+\mu_{24∕23}+\mu_{24∕24}\\ =\mu_{14∕13}+\mu_{14∕14}+\mu_{14∕23}+\mu_{14∕24}+\mu_{23∕13}+\mu_{23∕14}+\mu_{23∕23}+\mu_{23∕24}, \end{array}$$

for *H*_0 _: *γ*_1 _= 0,

(16) $$\begin{array}{c} \mu_{13∕13}+\mu_{13∕24}+\mu_{14∕13}+\mu_{14∕24}+\mu_{23∕13}+\mu_{23∕24}+\mu_{24∕13}+\mu_{24∕24}\\ =\mu_{13∕14}+\mu_{13∕23}+\mu_{14∕14}+\mu_{14∕23}+\mu_{23∕14}+\mu_{23∕23}+\mu_{24∕14}+\mu_{24∕23}, \end{array}$$

for *H*_0 _: γ_2 _= 0,

(17) $$\begin{array}{c} \mu_{13∕13}+\mu_{13∕14}+\mu_{14∕13}+\mu_{14∕14}+\mu_{23∕23}+\mu_{23∕24}+\mu_{24∕23}+\mu_{24∕24}\\ =\mu_{13∕23}+\mu_{13∕24}+\mu_{14∕23}+\mu_{14∕24}+\mu_{23∕13}+\mu_{23∕14}+\mu_{24∕13}+\mu_{24∕14}, \end{array}$$

for *H*_0 _: *I*_*αα *_= 0,

(18) $$\begin{array}{c} \mu_{13∕13}+\mu_{13∕23}+\mu_{14∕13}+\mu_{14∕23}+\mu_{23∕14}+\mu_{23∕24}+\mu_{24∕14}+\mu_{24∕24}\\ =\mu_{13∕14}+\mu_{13∕24}+\mu_{14∕14}+\mu_{14∕24}+\mu_{23∕13}+\mu_{23∕23}+\mu_{24∕13}+\mu_{24∕23}, \end{array}$$

for *H*_0 _: *I*_*αβ *_= 0,

(19) $$\begin{array}{c} \mu_{13∕13}+\mu_{13∕14}+\mu_{14∕23}+\mu_{14∕24}+\mu_{23∕13}+\mu_{23∕14}+\mu_{24∕23}+\mu_{24∕24}\\ =\mu_{13∕23}+\mu_{13∕24}+\mu_{14∕13}+\mu_{14∕14}+\mu_{23∕23}+\mu_{23∕24}+\mu_{24∕13}+\mu_{24∕14}, \end{array}$$

for *H*_0 _: *I*_*βα *_= 0,

(20) $$\begin{array}{c} \mu_{13∕13}+\mu_{13∕23}+\mu_{14∕14}+\mu_{14∕24}+\mu_{23∕13}+\mu_{23∕23}+\mu_{24∕14}+\mu_{24∕24}\\ =\mu_{13∕14}+\mu_{13∕24}+\mu_{14∕13}+\mu_{14∕23}+\mu_{23∕14}+\mu_{23∕24}+\mu_{24∕13}+\mu_{24∕23}, \end{array}$$

for *H*_0 _: *I*_*ββ *_= 0,

(21) $$\begin{array}{c} \mu_{13∕13}+\mu_{13∕24}+\mu_{14∕13}+\mu_{14∕24}+\mu_{23∕14}+\mu_{23∕23}+\mu_{24∕14}+\mu_{24∕23}\\ =\mu_{13∕14}+\mu_{13∕23}+\mu_{14∕14}+\mu_{14∕23}+\mu_{23∕13}+\mu_{23∕24}+\mu_{24∕13}+\mu_{24∕24}, \end{array}$$

for *H*_0 _: *J*_*αγ *_= 0,

(22) $$\begin{array}{c} \mu_{13∕13}+\mu_{13∕24}+\mu_{14∕14}+\mu_{14∕23}+\mu_{23∕13}+\mu_{23∕24}+\mu_{24∕14}+\mu_{24∕23}\\ =\mu_{13∕14}+\mu_{13∕23}+\mu_{14∕13}+\mu_{14∕24}+\mu_{23∕14}+\mu_{23∕13}+\mu_{24∕13}+\mu_{24∕24}, \end{array}$$

for *H*_0 _: *J*_*βγ *_= 0,

(23) $$\begin{array}{c} \mu_{13∕13}+\mu_{13∕14}+\mu_{14∕23}+\mu_{14∕24}+\mu_{23∕23}+\mu_{23∕24}+\mu_{24∕13}+\mu_{24∕14}\\ =\mu_{13∕23}+\mu_{13∕24}+\mu_{14∕13}+\mu_{14∕14}+\mu_{23∕13}+\mu_{23∕14}+\mu_{24∕23}+\mu_{24∕24}, \end{array}$$

for *H*_0 _: *K*_*γα *_= 0,

(24) $$\begin{array}{c} \mu_{13∕13}+\mu_{13∕23}+\mu_{14∕14}+\mu_{14∕24}+\mu_{23∕14}+\mu_{13∕24}+\mu_{24∕13}+\mu_{24∕23}\\ =\mu_{13∕14}+\mu_{13∕24}+\mu_{14∕13}+\mu_{14∕23}+\mu_{23∕13}+\mu_{23∕23}+\mu_{24∕14}+\mu_{24∕24}, \end{array}$$

for *H*_0 _: *K*_*γβ *_= 0, and

(25) $$\begin{array}{c} \mu_{13∕13}+\mu_{13∕24}+\mu_{14∕14}+\mu_{14∕23}+\mu_{23∕14}+\mu_{23∕23}+\mu_{24∕13}+\mu_{24∕24}\\ =\mu_{13∕14}+\mu_{13∕23}+\mu_{14∕13}+\mu_{14∕24}+\mu_{23∕13}+\mu_{23∕24}+\mu_{24∕14}+\mu_{24∕23}, \end{array}$$

for *H*_0 _: *L*_*γγ *_= 0, respectively. Each of these constraints is implemented with the EM algorithm as described above, which will lead to the MLEs of the genotypic values that satisfies equations (11) - (25), respectively. The critical thresholds for each of the tests (11) - (25) can be determined from simulation studies.

## Results

### A Worked Example

We use a real example of a forest tree to illustrate our multiallelic epistiatic QTL mapping method in an outbred population. The material was an interspecific F_1 _hybrid population between *Populus deltoides *(P_1_) and *P. euramericana *(P_2_). A total of 86 individuals were selected for QTL mapping. A genetic linkage map was constructed by using 74 SSR markers of segregating genotypes 12 × 34, which covers 822.35 cM of the whole genome and contains 14 linkage groups. The total number of roots per cutting (TNR) was measured and showed large variation in the hybrid population during the later development stage of adventitious rooting in water culture.

**Figure 1:**
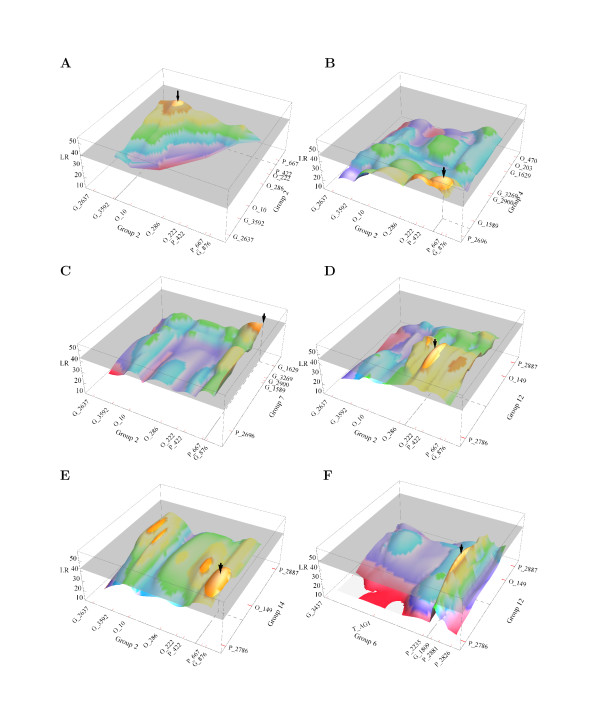
**The landscapes of log-likelihood ratio (LR) values testing the existence of two interacting QTLs controlling the total number of roots per cutting over different linkage groups**. **A**. one QTL from linkage group 2 interacting with the second QTL from linkage group 2. **B**. one QTL from linkage group 2 interacting with the second QTL from linkage group 4. **C**. one QTL from linkage group 2 interacting with the second QTL from linkage group 7. **D**. one QTL from linkage group 2 interacting with the second QTL from linkage group 12. **E**. one QTL from linkage group 2 interacting with the second QTL from linkage group 14. **F**. one QTL from linkage group 6 interacting with the second QTL from linkage group 12. In each case, the peak of the LR landscape (shown by an arrow) beyond the threshold surface (indicated in grey) shows the positions of two epistatic QTLs. The names and positions of markers at each group are indicated.

Through a systematic search over these linkage groups, the multiallelic espistatic model identifies six significant pairs of QTLs from different groups for TNR at the 5% significance level (Figure 1). The group × group-wide LR threshold for asserting that a pair of interacting QTLs exist was determined from 1000 permutation tests. Linkage group 2 has multiple regions that contain QTLs, which are located between markers L2_G_3592 and L2_O_10, markers L2_P_422 and L2_P_667, markers L2_P_667 and L2_G_876, and markers L2_O_286 and L2_O_222. These QTLs form five epistatic combinations by interacting with each other or with those on linkage groups 4, 7, 12 and 14 (Table 1). The sixth pair comes from linkage groups 6 and 12.

**Table 1:** Parameter estimates of interacting QTLs for root numbers in a full-sib family of poplars

Parameter	Estimate					
	L2 G 3592	L2 P 422	L2 P 667	L2 O 286	L2 P 422	L6 P 2235
QTL 1 Position	|	|	|	|	|	|
	L2 O 10	L2 P 667	L2 G 876	L2 O 222	L2 P 667	L6 G 1809
	L2 P 422	L4 P 2696	L7 G 3269	L12 P 2786	L14 P 2786	L12 P 2786
QTL 2 Position	|	|	|	|	|	|
	L2 P 667	L4 G 1589	L7 G 1629	L12 O 149	L14 O 149	L12 O 149
*μ*	2.0536	2.0578	2.0306	2.088	2.0438	2.0715
*α*_1_	-0.0081	0.0382	0.0113	-0.0462	0.0457	0.1275
*β*_1_	-0.1126	-0.1637	-0.2318	-0.181	-0.2119	0.0394
*γ*_1_	0.0452	0.1798	0.0907	0.1888	0.1785	0.1662
*α*_2_	0.0584	-0.0607	-0.0344	-0.1208	-0.0281	-0.0667
*β*_2_	-0.1898	-0.0096	-0.1207	0.0073	0.0758	0.0067
*γ*_2_	0.1276	-0.1329	0.1373	0.1029	0.1241	0.0435
*I*_*αα*_	-0.0638	-0.1602	-0.0113	-0.1002	0.1761	-0.0693
*I*_*αβ*_	0.0329	-0.1755	0.0945	-0.1029	-0.0044	0.1519
*I*_*βα*_	0.0535	-0.0064	0.0306	-0.2074	-0.0799	-0.1880
*I*_*ββ*_	0.1380	0.0480	-0.1335	-0.3300	0.0738	-0.1594
*J*_*αγ*_	-0.0498	0.0886	-0.0412	-0.0422	0.0613	0.0721
*J*_*βγ*_	0.0592	0.0309	0.0317	0.1105	-0.1096	0.0716
*K*_*γα*_	-0.0006	0.1228	0.054	-0.018	0.0165	0.2394
*K*_*γβ*_	-0.0928	-0.0063	-0.1124	-0.0905	-0.0433	-0.1011
*L*_*γγ*_	-0.0979	0.0886	-0.04	-0.1476	-0.0011	0.1952
*σ*^2^	0.187	0.1481	0.1809	0.0763	0.1549	0.0808
*LR*	40.9709	51.6811	46.4261	50.3592	47.6986	52.4637
*LR*_0.05_	39.6061	46.4006	42.7068	45.3719	42.5698	48.0733

Table 1 gives the estimates of genetic effect parameters for the six pairs of interacting QTLs. At QTLs on linkage group 2, parent *P. euramericana *tends to contribute unfavorable alleles to root number, as seen by many negative *β *values, although this parent shows a better rooting capacity than parent *P. deltoides*. At these QTLs, parent *P. deltoides *generally contributes a small-effect allele to root number, as seen by small *α *values. At the QTL on linkage group 6, this parent triggers a large positive additive effect. It is interesting to find that there are pronounced interactions between alleles from these two parents, as seen by large *γ *values, suggesting the importance of dominance in rooting capacity. In many cases, additive × additive epistatic effects are important, as indicated by many large *I *values. Our model can further discern which kind of additive × additive epistasis contribute. For example, the additive × additive epistasis between QTLs from linkage group 2 is due to the interaction between alleles from parent *P. euramericana*, while for QTL pair from linkage groups 2 and 14 this is due to the interaction between alleles from parent *P. deltoides*. The pattern of how the QTLs interact with each other in terms of additive × dominant, dominant × additive, and dominant × dominant epistasis can also be identified (Table 1).

### Monte Carlo Simulation

We performed simulation studies to investigate the statistical properties of the multiallelic epistatic model. We simulated a full-sib family of sample size 400, 800 and 2000 derived from two outcrossing parents. Two QTLs were assumed at different locations of a 100 cM-long linkage group with 6 even-spaced markers. Phenotypic values of a quantitative trait for each individual were simulated as the genotypic values at these QTLs plus normally distributed errors (scaled to have different heritabilities, 0.1 and 0.4). Genotypic values are expressed in terms of genetic actions and interactions with true values tabulated in Table 2.

**Table 2:** Parameter estimates and their standard errors of the multiallelic epistatic model for an outbred cross based on 1000 repeat simulations

		*H*^2 ^= 0.l			*H*^2 ^= 0.4		
			
Parameter	True Value	*N *= 400	*N *= 800	*N *= 2000	*N *= 400	*N *= 800	*N *= 2000
QTL 1 Position	30	29.64 (5.66)	29.90 (4.27)	29.86 (2.51)	30.00 (3.27)	30.04 (2.14)	29.99 (1.32)
QTL 2 Position	70	70.26 (5.73)	70.13(4.16)	70.04 (2.37)	70.07 (3.06)	70.03 (2.04)	69.96 (1.28)
*μ*	50.0	50.12 (3.02)	50.15 (2.08)	49.98 (1.25)	50.15 (1.26)	50.04 (0.82)	50.04 (0.51)
*α*_1_	2.0	2.03 (3.27)	1.95 (2.20)	2.02 (1.37)	2.11 (1.35)	2.07 (0.85)	2.03 (0.55)
*β*_1_	3.0	2.90 (3.33)	2.95 (2.21)	2.99 (1.39)	3.08 (1.33)	2.99 (0.90)	3.01 (0.54)
*γ*_1_	4.0	3.72 (3.61)	4.08 (2.52)	3.95 (1.50)	3.89 (1.47)	3.99 (0.98)	3.98 (0.58)
*α*_2_	-3.0	-3.11 (3.37)	-2.92 (2.11)	-2.99 (1.37)	-3.14 (1.34)	-3.04 (0.89)	-3.02 (0.53)
*β*_2_	1.0	1.06 (3.26)	1.02 (2.13)	0.96 (1.36)	1.01 (1.36)	0.98 (0.88)	1.01 (0.53)
*γ*_2_	-2.5	-2.62 (3.69)	-2.39 (2.44)	-2.57 (1.52)	-2.59 (1.42)	-2.48 (0.99)	-2.53 (0.60)
*I*_*αα*_	-2.0	-2.22 (3.62)	-2.30 (2.57)	-2.04 (1.58)	-2.15 (1.49)	-2.11 (0.95)	-2.02 (0.61)
*I*_*αβ*_	2.5	2.67 (3.64)	2.43 (2.44)	2.53 (1.52)	2.61 (1.47)	2.50 (0.94)	2.53 (0.59)
*I*_*βα*_	-3.0	-2.64 (3.61)	-3.10 (2.44)	-2.95 (1.53)	-2.94 (1.49)	-3.02 (0.95)	-2.98 (0.61)
*I*_*ββ*_	3.5	3.11 (3.48)	3.30 (2.47)	3.43 (1.51)	3.32 (1.47)	3.43 (0.95)	3.46 (0.60)
*J*_*αγ*_	-4.0	-3.82 (4.00)	-3.90 (2.77)	-3.91 (1.70)	-3.99 (1.56)	-3.97 (1.07)	-3.99 (0.67)
*J*_*βγ*_	-4.5	-4.07 (4.03)	-4.36 (2.66)	-4.47 (1.67)	-4.37 (1.50)	-4.41 (1.01)	-4.48 (0.65)
*K*_*γα*_	-2.0	-1.93 (4.10)	-2.02 (2.74)	-2.06 (1.67)	-2.12 (1.51)	-2.05 (1.06)	-2.01 (0.67)
*K*_*γβ*_	2.5	2.32 (4.12)	2.41 (2.68)	2.46 (1.68)	2.39 (1.46)	2.40 (1.02)	2.48 (0.64)
*L*_*γγ*_	-5.0	-4.42 (4.16)	-4.68 (3.07)	-4.88 (1.90)	-4.84 (1.69)	-4.90 (1.11)	-4.98 (0.73)
*σ*^2^	1334.3	1242.64 (99.80)	1286.12 (71.09)	1314.93 (43.50)			
*σ*^2^	222.4				206.83 (17.44)	214.62 (11.88)	219.40 (7.45)

Two QTLs are set at 30 cM and 70 cM in a chromosome of 100cM with 6 markers evenly spaced and the true parameters are set as in the second column.

It was found that the QTL positions can well be estimated using our model (Table 2). The additive effects at individual QTLs and additive × additive epistatic effects can be reasonably estimated even when a modest sample size is used for a modest heritability. The other genetic effect parameters, especially dominant × dominant epistatic effects, need a large sample size to be reasonably estimated especially when the heritability is low. Because of a large number of parameters involved, the outcrossing design requires much larger sample sizes than backcross or F_2 _designs.

## Discussion

The past two decades have seen a tremendous interest in developing statistical models for QTL mapping of complex traits inspired by Lander and Botestin's (1989) pioneered interval mapping [2,3,17,22-25]. However, model development for QTL mapping in outbred populations, a group of species of great environmental and economical importance [26], has not received adequate attention. Only a few publications are available to QTL mapping in outcrossing species [12,13]. In this article, we present a quantitative genetic model for studying the epistasis of multiallelic QTLs and a computational algorithm for estimating and testing epistatic interactions.

The central issue of QTL mapping for outcrossing populations is how to model genetic actions and interactions between multiple alleles at different QTLs. Traditional quantitative genetic models have been developed for biallelic genetic effects [16] and their extension to multiallelic cases have not been clearly explored. This study gives a first attempt to characterize epistatic interactions between multiallelic QTLs that pervade outcrossing populations. We partition additive effects at each QTL into two subcomponents based on different parental origins of alleles. Similarly, we partition the additive × additive epistasis into four different subcomponents, the additive × dominant epistasis into two subcomponents, and the dominant × additive epistasis into two subcomponents based on the interactions of alleles of different parental origins. These subcomponents have unique biological meanings because they are derived from distinct parents. In practice, hybridization is made between two genetically distant parents, thus an understanding of each of these subcomponent helps to study the genetic basis of heterosis.

We tested the new multiallelic epistasis model through simulation studies. In general, because of a number of parameters involved, a larger sample size is required to obtain reasonably precise estimation for QTL mapping in outcrossing populations. According to our experience, the increased heritability of traits by precise phenotyping can improve parameter estimation and model power than augmented experiment scales. We recommend that more efforts are given to field management that can improve the quality of phenotype measurements than experimental size. By analyzing a real data set from a poplar genetic study, the new model has been well validated. It is interesting to find that interactions between alleles from different poplar species contribute substantially to rooting capacity from cuttings, larger than genetic effects of alleles that operate alone. This result may help to understand the role of dominance in mediating heterosis.

## Conclusions

We have developed a statistical model for mapping interactive QTLs in a full-sib family of outcrossing species. By capitalizing on traditional quantitative genetic theory, we define epistatic components due to interactions between two outcrossing multiallelic QTLs. An algorithmic procedure was derived to estimate all types of outcrossing epistasis and test their significance in controlling a quantitative trait. Our model provides a useful tool for studying the genetic architecture of complex traits for outcrossing species, such as forest trees, and fill a gap that occurs in genetic mapping of this group of important but underrepresented species.

## Authors' contributions

CT derived the model and performed computer simulation and data analysis. BZ and MX collected the data from poplar hybrids. ZW and JS participated in simulation studies. XP participated in model design and result interpretation. MH conceived of the experiment. RW developed the model and algorithm, coordinated simulation and data analysis, and wrote the paper. All authors have read and approved the final manuscript.
